# Spontaneous Intracerebral Haemorrhage in a Child

**Published:** 2012-03-01

**Authors:** Amit Agrawal, Vikram Jeet Singh Dhingra

**Affiliations:** Department of Neurosurgery, MM Institute of Medical Sciences and Research, Mullana (Ambala), India.; Department of Surgery, MM Institute of Medical Sciences and Research, Mullana (Ambala), India.

**Keywords:** Spontaneous intracerebral haemorrhage, Intracerebral haematoma

## Abstract

Spontaneous intracerebral haemorrhage (SICH) is a rare occurrence in children, with different aetiological factors, clinical characteristics and prognosis. A 14 year male child had sudden onset of headache associated with multiple vomiting. Magnetic resonance imaging showed deep seated intracerebral haematoma. Haematoma was evacuated successfully and child recovered without deficits. A high index of suspicion is necessary for the diagnosis of spontaneous intracerebral haemorrhage in children.

## INTRODUCTION

Spontaneous intracerebral haemorrhage is a rarely reported in children. Its aetiology is different from that of adults and distinct clinical characteristics and prognosis. If not suspected these cases can be misdiagnosed initially as meningitis or common cold [1-4]. Herein a case of SICH is presented to highlight recognition of this condition. 

## CASE REPORT

A 14 year old male child had sudden onset of headache one and half month back which was associated with multiple episodes of vomiting. He was treated at local hospital and headache was reduced in intensity after oral analgesics. There was no history of fever, seizures or focal neurological deficits. His general and systemic examination was unremarkable. Higher mental functions were normal and there was no focal neurological deficit. Fundus showed bilateral early papilloedema. Blood investigations were normal. He was investigated with magnetic resonance imaging (MRI) which showed evidence of left basal ganglionic haematoma with mass effect and midline shift (Fig. 1,2). He underwent left frontal craniotomy and haematoma was approached through the middle frontal gyrus as it was approaching to the surface in that region. There was thin capsule containing altered blood which was removed completely. Histopathology showed organizing blood clot and there was no evidence of tumor cells or any abnormal vessels (Fig. 3). Child is doing well and there are no neurological deficits.

**Figure:**
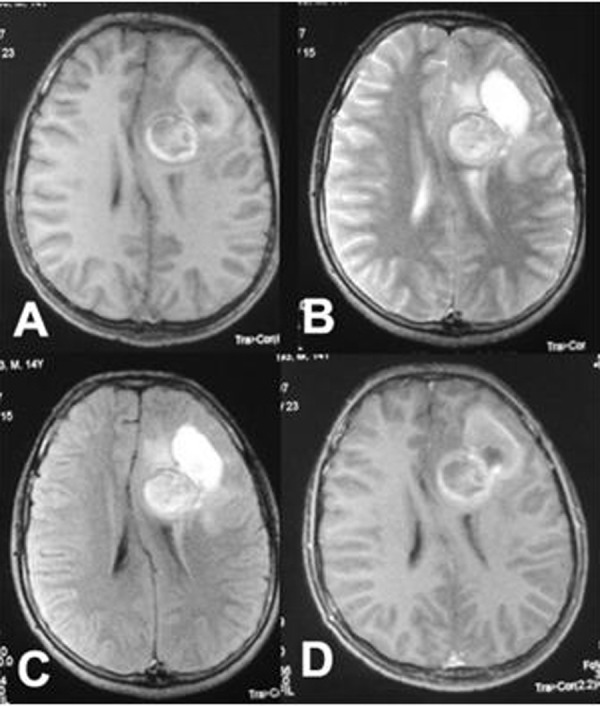
Figure 1: MRI axial section showing well defined lesion in left basal ganglion with evidence of haemorrhage, (A) T1, (B) T2, (C) FLAIR images and (D) minimal enhancement after contrast administration.

**Figure:**
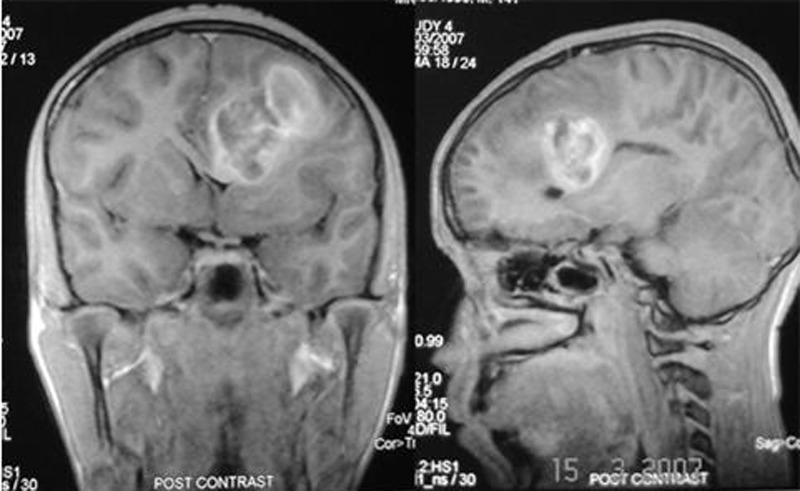
Figure 2: Contrast MRI coronal and sagittal sections showing more details of the lesion; note the mass effect and distortion of corpus callosum.

**Figure:**
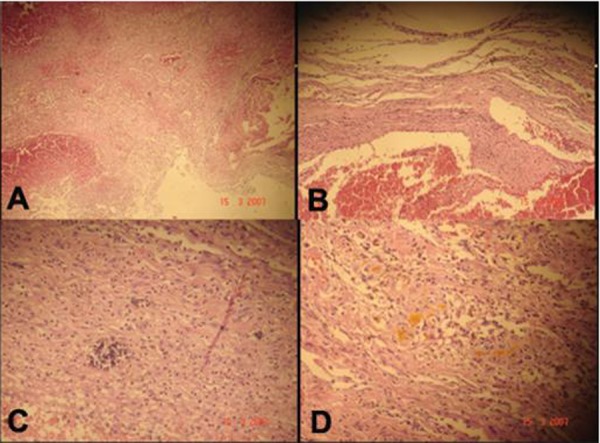
Figure 3: Photomicrograph showing features of organized haematoma (A) 20x, (B) 40x, (C) evidence of calcification and (D) haemosiderin deposits.

## DISCUSSION

Spontaneous intracerebral haemorrhage more commonly affects male children. The leading cause of SICH is arteriovenous malformation. Other causes include haematologic or coagulation disorders, serious liver disorder due to alpha-l-antitrypsin deficiency; bleeding from an undiagnosed tumor and sometime one may not be able to find the aetiology. SICH usually present with a sudden onset of headache that may be associated with vomiting and altered consciousness. Magnetic resonance imaging scans is the most important non-invasive modality for the investigation. Cerebral angiography can diagnose arteriovenous malformations. Laboratory examinations are directed to rule out the other causes of bleeding i.e. bleeding disorders or haematological malignancies [1-4].

Management of critical SICH consists in preventing or treating cerebral hypertension and seizures. Prompt excision of the haematoma improves the outcome as in present case. Patients with poor neurological status at the time of admission and SICH located at brain stem, cerebellum, and multiple subcortical areas have higher mortality rates. Intraparenchymatous haematomas in children have a high mortality and many sequelae. A high index of suspicion is necessary for the diagnosis of spontaneous intracerebral haemorrhage in children as the incidence of SICH is very low and presenting symptoms may be non-specific [2-4].

## Footnotes

**Source of Support:** Nil

**Conflict of Interest:** None declared
